# The Tragic Tale of Wittenberg

**Published:** 1916-05-06

**Authors:** 


### THE TRAGIC TALE OF WITTENBERG.

### Shall there be Medical Reprisals?

Certain incidents of the war have not unnaturally given rise to a cry for an application to the enemy of the methods which in particular directions he has put in operation against ourselves, and the claim has had the endorsement of some names which enjoy general respect. Hardly anyone in this country would urge this argument in reference to all the practices which are placed to the discredit of our enemies, and many even of those who advocate reprisals in special cases take this stand with regret and, as they believe, with the object of protecting our own people. It is not within our scope to discuss the general question, but there is just now a proposal to adopt one form of the policy by the corporate action of the medical profession, and individual members of the profession will have to make up their minds on the point in one direction or the other.

The proposal to which allusion is here made, we need hardly say, has been prompted by the shameful and tragic tale of Wittenberg. Within this story are acts of medical practitioners which no words can fully characterise, and the blackness of these seems all the darker by contrast with the simple and steadfast devotion to duty which was practised as a matter of course by our own men. The record both in one direction and the other stands, and it carries its own message. The suggestion is now made that, with a view to mark our detestation of the callous and brutal indifference and cowardice of certain of the medical officers responsible for the camp at Wittenberg, the names of all German practitioners which stand on the lists of our medical and professional societies shall be erased. It is not improbable that this proposal may attract considerable support, but for our own part we do not welcome it. In the first place, the proceeding contemplated is not a very brave one, seeing that the penalty, such as it is, is to be inflicted on men who are unable to retaliate. Nor does it satisfy what is usually regarded as fair play, for it condemns those who cannot be heard in their defence. Still again, the punishment has a vicarious quality, and is made to fall, not on the undoubtedly guilty but on some who may at least possibly be innocent. Is it for a moment credible that on the facts as stated there is any country in the world in which the medical profession as a whole would fail to pass an adverse judgment? Should there be such a country, the explanation doubtless is, not approval of the happenings but disbelief of the record. In time there may come the opportunity for the exposure and punishment of the real criminals, and here justice may exact its full measure with the concurrence of others than those who cannot now escape the influence of national enmities. What is now proposed is neither very forcible nor very dignified. It will, if adopted, have no practical value, and instead of the stern features of justice it will be apt to present to outsiders the characters of a small and angry spitefulness. When the true authors of the crime can be made to suffer for what they have done, few, if any, voices will plead for mercy, but in the meantime we shall do well to be silent and resolved. To clamour for a victim is hardly becoming to those who practise the art of medicine.

